# Microstructure Analysis and Effects of Single and Mixed Activators on Setting Time and Strength of Coal Gangue-Based Geopolymers

**DOI:** 10.3390/gels8030195

**Published:** 2022-03-21

**Authors:** Xiaoyun Yang, Yan Zhang, Cheng Lin

**Affiliations:** 1 College of Energy and Transportation Engineering, Inner Mongolia Agricultural University, Hohhot 010018, China; ycyangxiaoyun@emails.imau.edu.cn; 2 Department of Civil Engineering, University of Victoria, Victoria, BC V8P 5C2, Canada; chenglin918@uvic.ca

**Keywords:** coal gangue, geopolymer, setting time, strength, microstructure

## Abstract

Geopolymer is a green non-metallic material with high strength and favorable properties in resistance to corrosion, fire, and high temperature, which makes it a potential substitute for Portland cement. The existing studies have primarily focused on the preparation of geopolymers using silico-alumina materials such as fly ash, red mud, metakaolin, volcanic ash, and blast furnace slag to develop geopolymers. This study explores the potential of using ultrafine calcined coal gangue and ground granulated blast furnace slag to develop a new geopolymer with the activation of a single activator (sodium hydroxide) or mixed activator (sodium hydroxide, liquid sodium silicate, and desulfurization gypsum). The setting time and strength of the geopolymers were investigated, followed by the mineral, functional groups, microstructure, and elements analyses using X-ray diffraction, Fourier transform infrared diffraction, scanning electron microscope, and energy dispersive spectrometer to elucidate the effect of different activators on geopolymers. The results showed that the optimum molarity of NaOH single activator was 2 mol/L, the initial setting time and final setting time were 37 min and 47 min, respectively, and the compressive and flexural strengths at 28 days were 23.2 MPa and 7.5 MPa. The optimal mixing ratio of the mixed activator was 6% desulfurization gypsum, 0.6 Na_2_SiO_3_ modulus, and 16% SS activator; the initial setting time and final setting time were 100 min and 325 min, respectively, and the compressive and flexural strengths at 28 days were 40.1 MPa and 7.8 MPa. The coal gangue geopolymers were mainly C–A–S–H, N–A-S-H, and C–N–A–S–H gels. The mixed activator tended to yield higher strengths than the single activator, the reason is that the hydration reaction was violent and produced more gels. Meanwhile, the relation between setting time and activator and the relation between strength and activator were also obtained, which provide theoretical support for predicting the setting time of coal gangue base polymer and the ratio of alkali activator for geopolymers with a certain strength.

## 1. Introduction

Geopolymer is a high-performance inorganic non-metallic material, possessing advantageous physical and chemical properties such as high strength, corrosion resistance, and high temperature resistance [1,2,3]. As a green and environmentally friendly material, geopolymer has been applied in engineering. For example, geopolymer was used in turning node, apron, and taxiway pavements at Brisbane West Wellcamp Airport, Australia [4]. In terms of building materials, geopolymers are used to make floor slabs, roof tiles, etc. [5,6], and have advantages in 3D building material printing and carbon dioxide sequestration [7,8]. The formation of a geopolymer gel requires silicon and aluminum rich materials activated in an alkaline solution. Coal gangue, a by-product of coal production, accounts for approximately 10–15% of the total coal production. In the coal washing process, the residential gangue accounts for approximately 30% of the washed clean coal. As a solid waste, coal gangue is often piled in the open field, which can contaminate the surrounding environment, e.g., dust, toxic gas, heavy metal leaching, and acidic pollution [9,10,11]. Coal gangue contains a large amount of silica and alumina is a desirable raw material that can be used to produce geopolymer gel [12,13,14,15]. The use of coal gangue in the production of geopolymers can effectively solve the problem of environmental pollution. Blast furnace slag (BFS) is also an industrial solid waste, generated during cast iron smelting. Ground granulated blast furnace slag (GGBFS) is a powder of blast furnace slag that has been dried and ground to a considerable fineness and meets the corresponding activity index. Like coal gangue, GGBFS is also a favorable raw material for geopolymer [16,17]. Moreover, it can accelerate the geopolymerization and thus allows for rapid early strength development. However, due to its wide range of applications and limited supply, BFS is cost ineffective in practice. In contrast, coal gangue is abundant, and its cost for preparing geopolymers is low. As such, if the coal gangue can be used to partially replace BFS, it would significantly reduce the material cost while rendering tangible environmental benefits.

The activation effect of the geopolymer is related to the reactivity of the solid precursor, the type of the activator, and the activated method. The precursor materials are mainly fly ash, GGBFS, red mud, coal gangue, and other materials containing more silicon and aluminum elements, and their reactivity is related to the content of amorphous silicon, aluminum, and calcium elements and specific surface area. To improve the reactivity of raw materials, the commonly used methods are mechanical grinding and calcination. Mechanical grinding is a physical method of mechanically breaking or grinding raw materials using crushers, pulverizers, or ball mills. For example, coal gangue powder with particle size less than 80μm has better reactivity. This process results in solid crystallization and induces changes to the materials’ physical and chemical properties. Calcination belongs to thermal activation, and the reactivity of raw materials is increased by calcination. For raw coal gangue, its reactivity is relatively weak; however, this can be enhanced after high temperature treatment [13,18]. Research shows that calcination at a temperature of 700 °C for 2 h would transform coal gangue into amorphous metakaolin with strong activity [19]. Adding an activator to the raw material is a chemical activation method. Chemical activation relies on the alkali metal hydroxide (NaOH or KOH), alkali metal silicate (Na_2_SiO_3_ or K_2_SiO_3_), alkali metal carbonate (Na_2_CO_3_), and alkali metal sulphate (Na_2_SO_4_) to trigger geopolymerization [20,21,22]. 

In this research, the coal gangue was pretreated by mechanical (pulverizing) and thermal activation (calcining) methods, and then the coal gangue and slag were activated by different alkali activators (NaOH activator, NaOH + Na_2_SiO_3_ + desulfurized gypsum activator) to prepare the coal gangue-based geopolymer. By studying the effects of different activators on the solidification time and strength, the equations of the activator parameters with the solidification time and strength were obtained, so that the solidification time and the proportion of alkali activator at the expected strength could be predicted quickly. Meanwhile, the mineral, morphology, and functional group changes of the geopolymer gels were analyzed by using X-Ray diffraction (XRD), Fourier transform infrared spectroscopy (FTIR), scanning electron microscopy (SEM), and energy dispersive spectroscopy (EDS).

## 2. Results and Discussion

This section discusses the results of changes in appearance, setting time, and compressive and flexural strength of the proposed geopolymers, focused on the effects of single and mixed activators. The material composition, functional group changes, morphologies, and elements of different geopolymers were analyzed.

### 2.1. Appearance Observation

**Figure 1:**
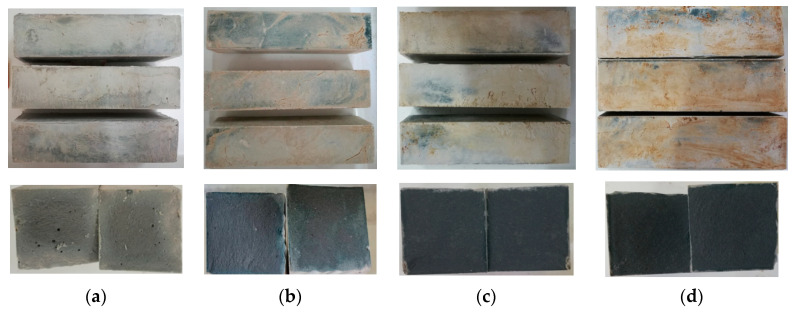
SH geopolymer and cross section (**a**) SH3, (**b**) SH8, (**c**) SH13, (**d**) SH18.

**Figure 2:**
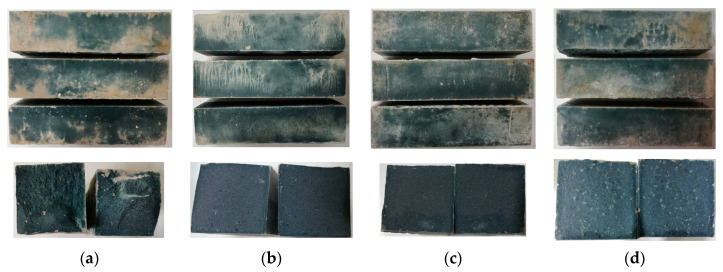
SSG geopolymer and cross section. (**a**) SSG1, (**b**) SSG5, (**c**) SSG9, (**d**) SSG13.

Figure 1 and Figure 2 show the appearance and section of SH geopolymer and SSG geopolymer cured for 28 days. The appearance color of SH was white and blue after 28 days of curing. It can be seen from the cross-sectional view that the more GGBFS powders were added, the inside appeared blue, and the formed material was denser. The appearance of SSG geopolymer was white-blue, but it was darker than SH, and its cross-section was blue. In addition, a small amount of desulfurized gypsum that had not participated in the reaction could be seen in the formed material.

### 2.2. Effects of Single Activator

#### 2.2.1. Setting Time of SH Geopolymer

**Figure 3:**
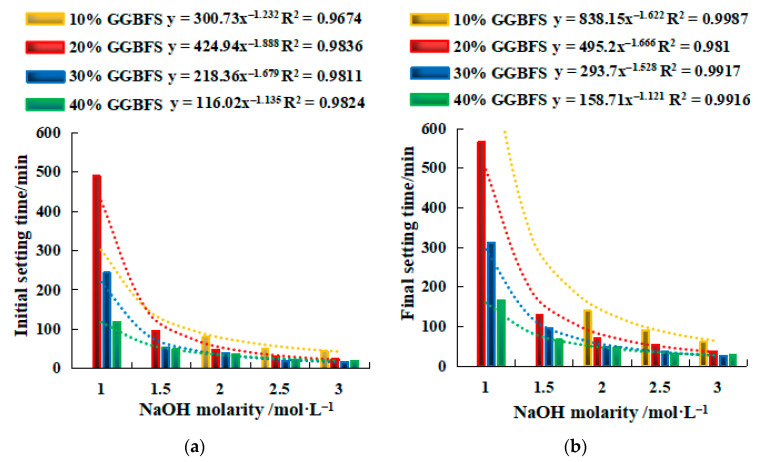
Relationship between NaOH molarity and setting time. (**a**) Initial setting time. (**b**) Final setting time. $y={\mathrm{ax}}^{-b}$, where y is the setting time, min. x is the NaOH molarity, 1 ≤ x ≤ 3 mol/L. a and b are constants. The value of a is related to the GGBFS dosage, and a value decreases with the increase in GGBFS content. b is between 1 and 2.

Figure 3 shows the relationship of NaOH molarity and setting time. The setting time decreased with the increase in GGBFS dosage and NaOH molarity. The effect of GGBFS dosage on setting time was greater than that of NaOH molarity. Too small a GGBFS dosage will greatly prolong the setting time, resulting in poor excitation effect. In addition, the NaOH molarity needs to be greater than a certain value to be activated. For example, for a geopolymer with 10% GGBFS, the molarity of NaOH must be greater than 1.5 mol/L to activate. The minimum NaOH molarity of 20–40% GGBFS should be greater than 1 mol/L. The initial setting times of SH10, SH15, and SH20 (3 mol/L NaOH and the 20–40%GGBFS) were only 16.9%, 6.6%, and 4.9% of that of SH16, SH11, and SH16 (1 mol/L NaOH and the 20–40% GGBFS), respectively. Meanwhile, the initial setting time of SH10, SH15, and SH20 was less than 30 min, and the longest final setting time was only 37 min. From the trend line in the figure, the relationship between setting time and NaOH molarity was highly consistent with a power function, which shows that after reaching to activate concentration, NaOH molarity slightly increased, setting time there would be a steep fall in, and the steep fall in degree decreased with the increase in dosage of GGBFS. Through the equations in Figure 3, the setting time of the geopolymer can be predicted at the same ratio of UCCG and GGBFS.

#### 2.2.2. Strength of SH Geopolymer 

**Figure 4:**
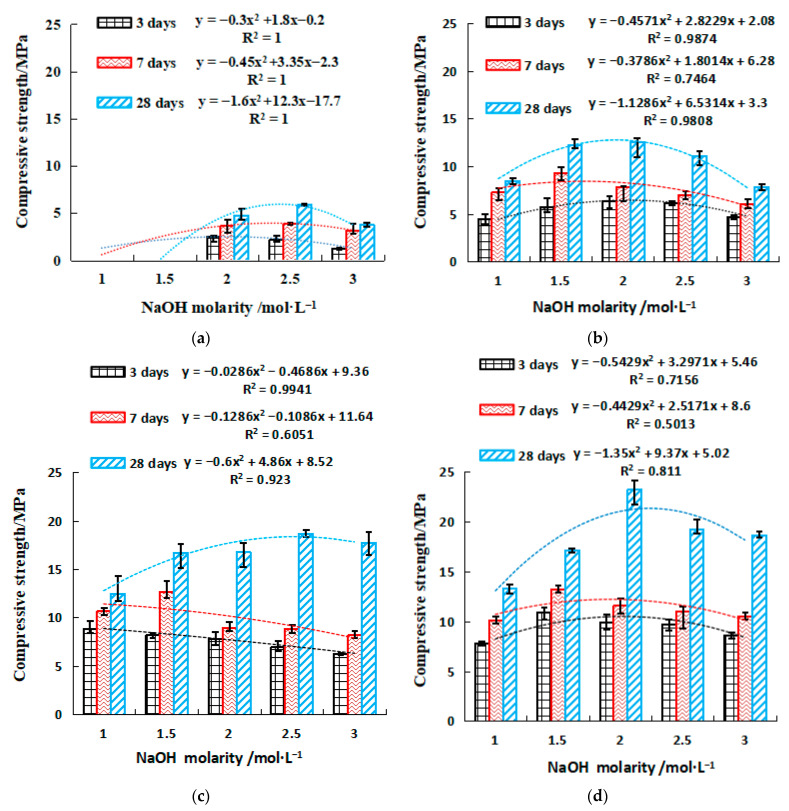
Compressive strength of NaOH single activator. (**a**) 10% GGBFS, (**b**) 20% GGBFS, (**c**) 30% GGBFS, (**d**) 40% GGBFS.

**Figure 5:**
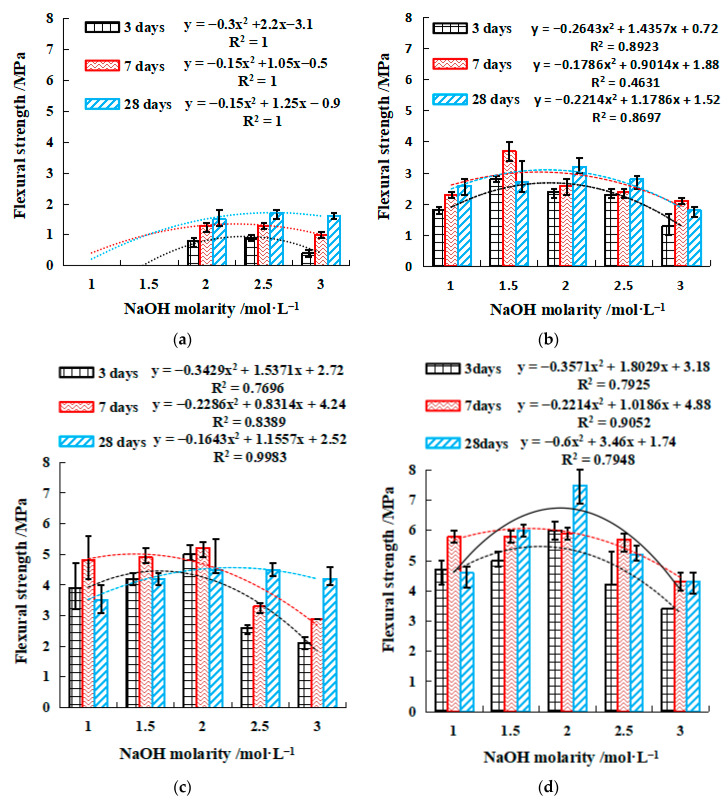
Flexural strength of single activator. (**a**) 10% GGBFS, (**b**) 20% GGBFS, (**c**) 30% GGBFS, (**d**) 40% GGBFS. $y={\mathrm{ax}}^{2}+\mathrm{bx}+c,$ where y is the compressive or flexural strength, MPa. x is the NaOH molarity, 1 ≤ x ≤ 3, mol·L^−1^. a, b, and c are coefficients.

Figure 4 and Figure 5 show the compressive and flexural strengths of the geopolymers with varied concentrations of NaOH, respectively. Both figures display that the strengths gradually increased with curing time within 28 days, and a higher strength growth rate was found between 3 and 7 days. It is also found that both compressive and flexural strengths appeared to consistently peak at approximately 2 mol/L NaOH for the range of 1 to 3 mol/L investigated. This indicates that NaOH accelerated the strength development of geopolymer when its concentration was relatively low. However, the strength development was suppressed at a higher concentration of NaOH. The increase in GGBFS led to a growth of the strengths. With only 10% GGBFS, the excitation of the geopolymer requires a larger alkali concentration, and the initial excitation concentration was 2 mol/L NaOH, the 28-day maximum compressive and flexural strength of SH4 (2.5 mol/L NaOH) was only 5.9 MPa and 1.7 MPa, respectively (Figure 4a and Figure 5a). However, the 28-day compressive and flexural strength of 40% GGBFS was 3.3–4.9 times and 2.7–5.0 times that of 10% GGBFS. It is worth noting that GGBFS dosage was limited to 40% as too large an addition of GGBFS would not be cost-effective. SH18 (40% GGBFS, 2 mol/L NaOH) has the highest compressive and flexural strength, 23.2 MPa and 7.5 MPa, respectively (Figure 4d and Figure 5d). Meanwhile, the strength–NaOH molarity curves followed a quadratic relationship. The molarity of NaOH required to achieve the corresponding compressive strength or flexural strength can be easily calculated by the equations in Figure 4 and Figure 5, to quickly configure an effective activator. 

### 2.3. Effects of Mixed Activator

#### 2.3.1. Setting Time of SSG Geopolymer

**Figure 6:**
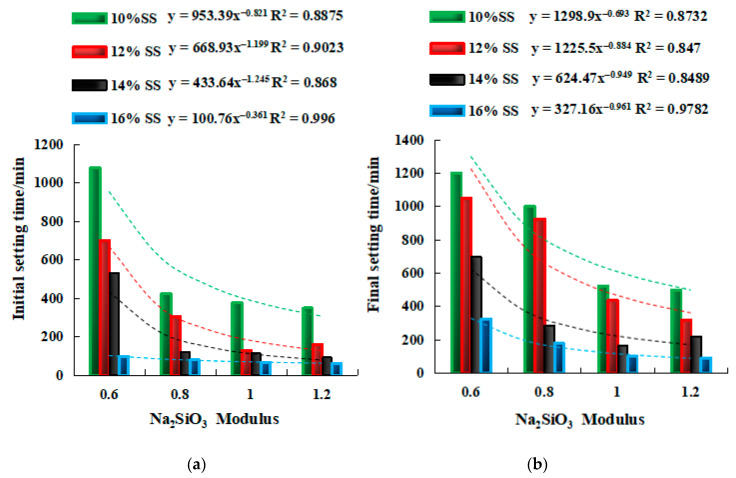
The relationship of setting time and modulus. (**a**) Initial setting time. (**b**) Final setting time. $y={\mathrm{ax}}^{2}+\mathrm{bx}+c,$ where y is the setting time, min. x is the Na_2_SiO_3_ modulus, 0.6 ≤ x ≤ 1.2. a, b, and c are constants.

In Figure 6, with the increase in SS alkali content, the setting time shows a decreasing trend, and the setting time of 16% SS was the shortest. For the modulus, an increase in the modulus also accelerated the reduction in the setting time. The initial setting time of SSG4, SSG8, SSG12, and SSG16 (M1.2) is only 32.4%, 23%, 17.5%, and 61% of that of SSG1, SSG5, SSG9, and SSG13 (M0.6), respectively. In 10% SS~14% SS, with the increase in modulus, the tendency of setting time to decrease was faster. The initial setting time of 16% SS was less than 100 min, and the final setting time was less than 325 min. The relationship between Na_2_SiO_3_ modulus and setting time fit a power function. The geopolymer setting time can be predicted exactly from the equations in Figure 6.

**Figure 7:**
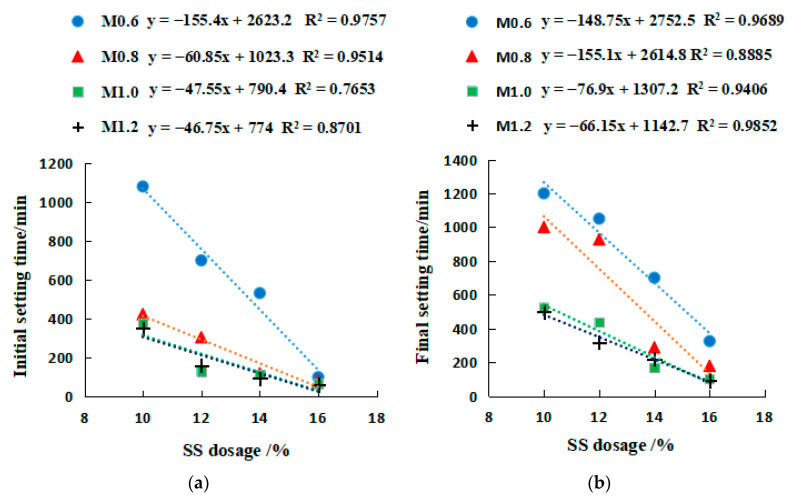
The relationship of setting time and SS dosage. (**a**) Initial setting time. (**b**) Final setting time. y=−ax+b, where y is the setting time, min. x is SS activator dosage, 10 ≤ x ≤ 16%. a and b are constants.

Figure 7 describes that under the same modulus, the setting time gradually decreased with the increase in SS alkali content, and there is a linear relationship between the setting time and SS alkali content. Therefore, the equations in Figure 7 can be used to accurately predict the setting time of samples with the same modulus.

#### 2.3.2. Strength of SSG Geopolymer 

**Figure 8:**
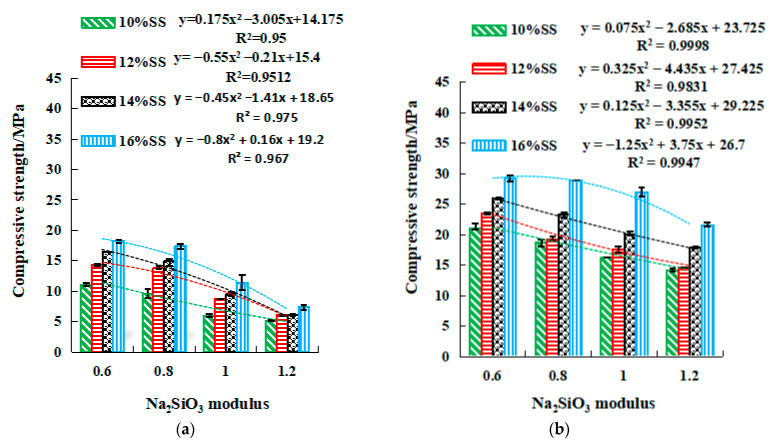

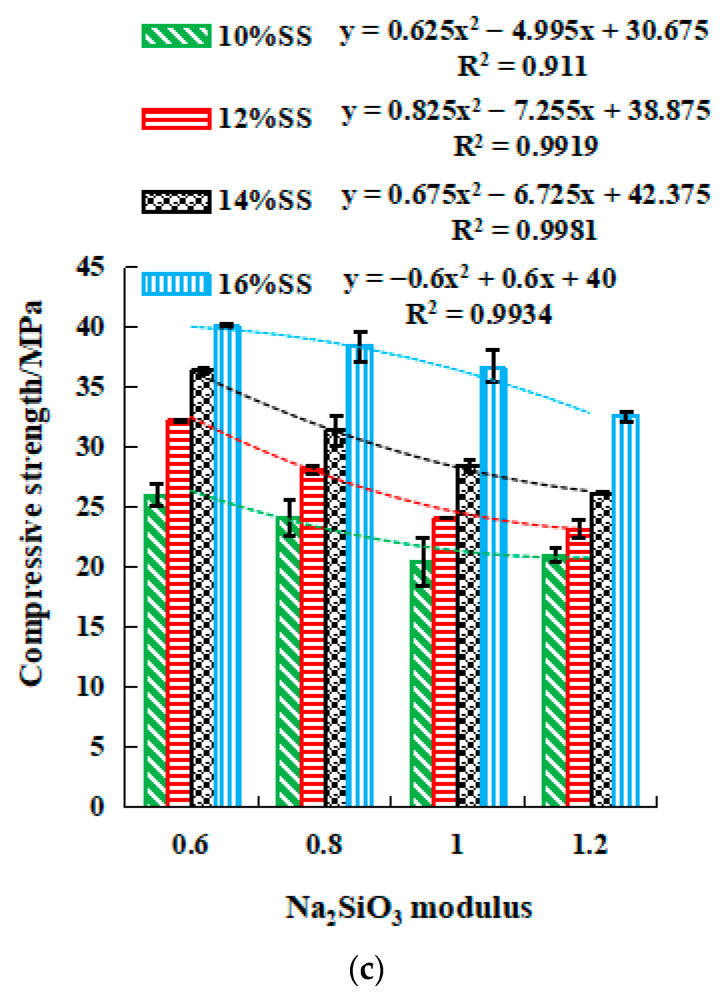
The relationship between modulus and compressive strength. (**a**) 3 days, (**b**) 7 days, (**c**) 28 days.

**Figure 9:**
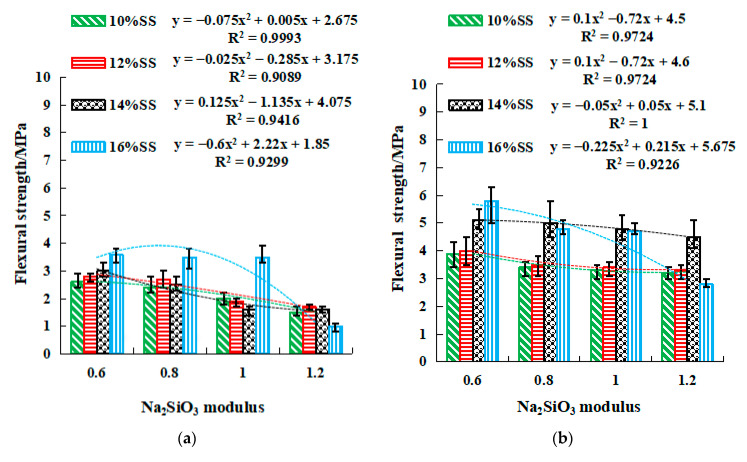

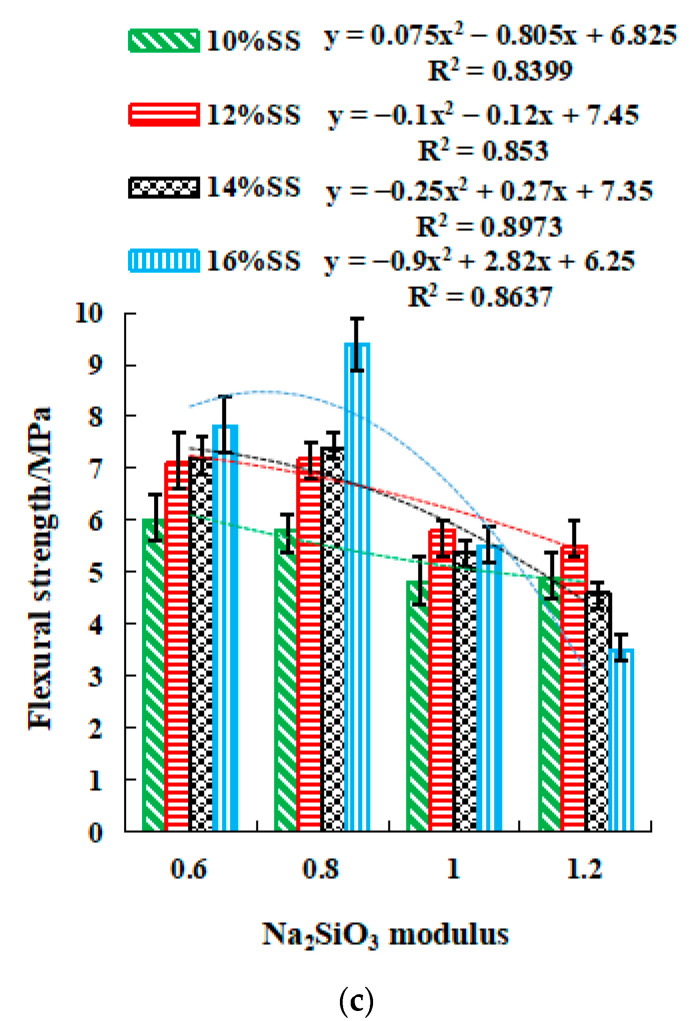
The relationship between modulus and flexural strength. (**a**) 3 days, (**b**) 7 days, (**c**) 28 days. $y={\mathrm{ax}}^{2}+\mathrm{bx}+c$, where y is the compressive or flexural strength, MPa. x is the Na_2_SiO_3_ modulus, and 0.6 ≤ x ≤ 1.2, mol L^−1^. a, b, and c are coefficients.

Figure 8 and Figure 9 show the relationship between Na_2_SO_3_ modulus and strengths, along with which the quadratic fit is also given. Both figures demonstrate that both compressive and flexural strengths reached the maximum when SS content and the modulus were equal to 16% and 0.6, respectively. The 3-day, 7-day, and 28-day compressive strength of the geopolymer with 0.6 modulus were approximately 2.5, 1.5, and 1.3 times that of the geopolymer with 1.2 modulus (Figure 8). The flexural strength for 0.6 modulus was 2.2, 1.4, and 1.7 times that for 1.2 modulus (Figure 9). This result reinforces the previous observation that the strength development was enhanced at a low modulus at the early stage. In this stage, an SSG13 geopolymer with high strength was prepared, and its compressive and flexural strength could reach 40.1 MPa and 7.8 MPa after curing for 28 days. Meanwhile, the relationship between strength and the modulus height conform to the quadratic function equation; according to the equations in Figure 8 and Figure 9, the Na_2_SiO_3_ modulus at a certain strength can be selected accurately.

**Figure 10:**
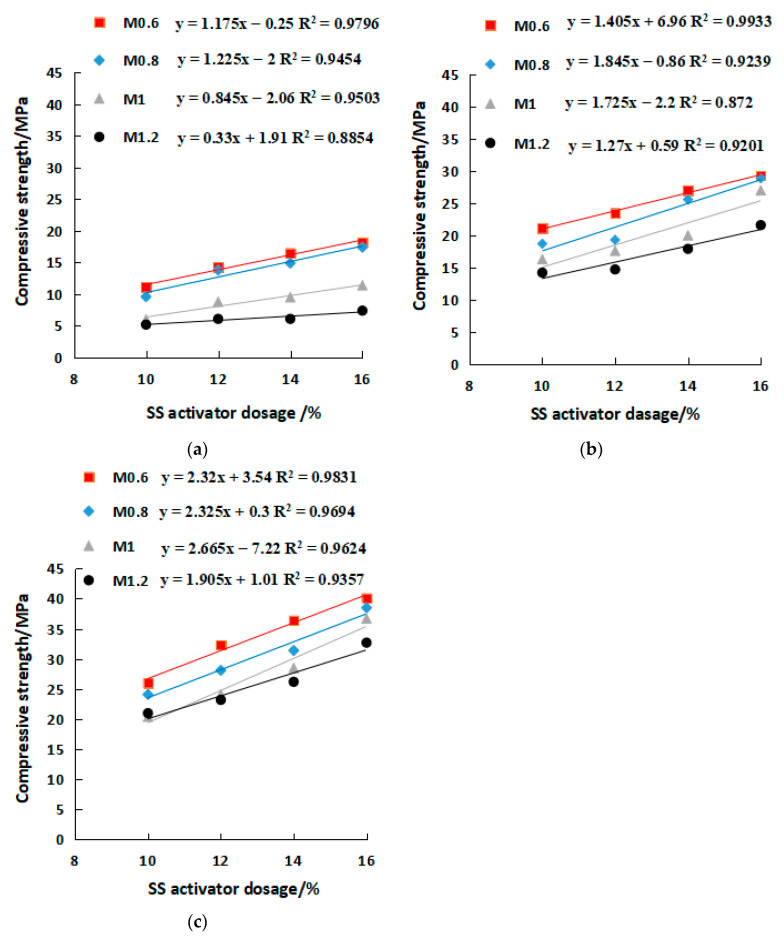
Relationship between compressive strength and SS activator dosage. (**a**) 3 days, (**b**) 7 days, (**c**) 28 days.

**Figure 11:**
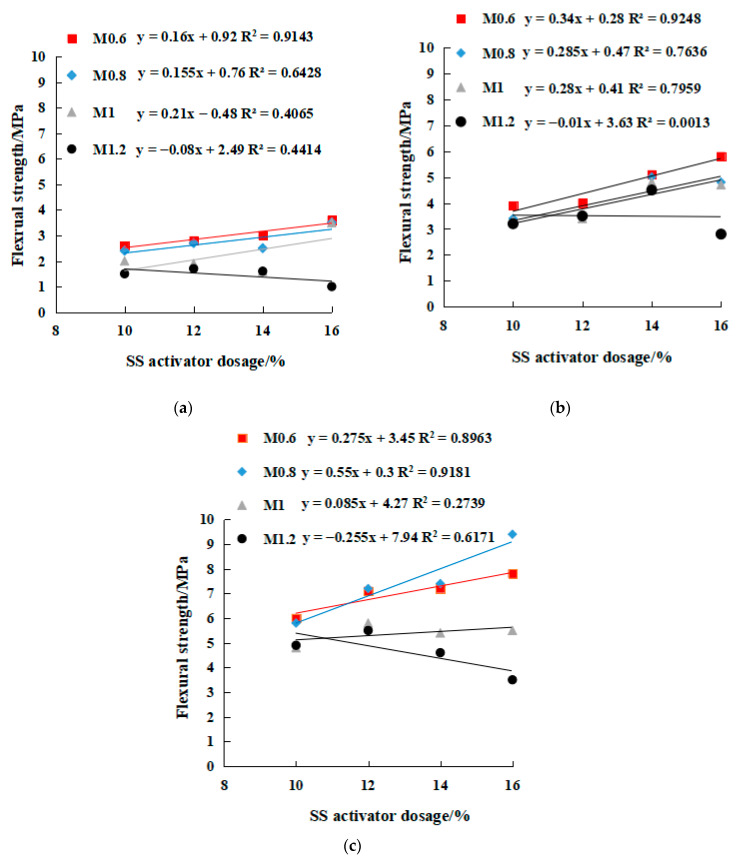
Relationship between flexural strength and SS activator dosage. (**a**) 3 days, (**b**) 7 days, (**c**) 28 days. y=ax+b, where y is the compressive or flexural strength, MPa. x is SS activator dosage, and 10 ≤ x ≤ 16%. a and b are constants.

Figure 10 and Figure 11 show the variations of compressive and flexural strengths with SS content. The increase in 3-day compressive strength due to the rise of SS from 10% to 16% ranged between 42.3% and 90% (Figure 10a), while the 7-day and 28-day compressive strength increase were in a range of 38.8–65.6% and 54.2–79.9%, respectively (Figure 10b,c). Therefore, increasing the SS alkali content in the early stage would help improve the compressive strength. In contrast, the trend of flexural strength with SS content did not follow a consistent pattern well, only the low modulus (M0.6–M0.8) R^2^ approached 1. The SS content of M0.6 and M0.8 increased from 10% to 16%, and the flexural strength of 3 days, 7 days, and 28 days increased by 38.4%, 48.7%, and 30%, respectively (Figure 11). Overall, the SS was beneficial for the strength development in geopolymers. Strength and alkali content are in line with a linear relationship. The R^2^ of the compressive strength is closer to 1, and the correlation is stronger than the flexural strength. In addition, the correlation coefficient R^2^ with a lower modulus is closer to 1 (such as M0.6). The equations in Figure 10 and Figure 11 can predict the alkali content of the corresponding activator at a certain strength through the relationship between alkali content and strength.

## 3. Characterization

The 28-day SH18 and SSG13 samples were selected for mineral and microstructural analyses using XRD, FTIR, SEM, and EDS -microscopic tests. Through these tests, the mineral composition, functional groups, and microscopic morphology of the two geopolymers were compared.

### 3.1. XRD Tests

**Figure 12:**
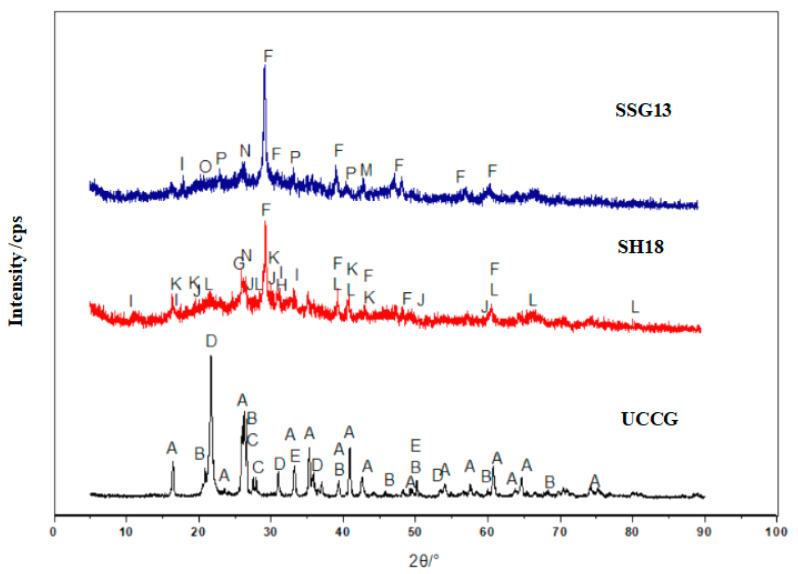
XRD patterns of UCCG and geopolymer. A: Sillimanite, B: Quartz, C: Kyanite, D: Cristobalite, E: Hematite, F: Calcite, G: Analcime, H: Gehlenite, I: zoisite, J: Gonnardite, K: Thomsonite, L: Mesolite, M: Nepheline, N: kyanite, O: Gypsum, P: Anorthite.

Figure 12 shows the XRD patterns of the UCCG powder and the two types of geopolymers. Mineral composition was analyzed using Jade 6.0 software. The main components of the UCCG were sillimanite, quartz and cristobalite. After being activated by the alkaline solution, the sillimanite and cristobalite peaks almost disappeared, indicating that they were involved in geopolymerization. The positions of the peak intensity of minerals in SH18 and SSG13 geopolymers were almost identical, but the peak intensities were different. The main reason is that the minerals produced in single activator and mixed activator samples were the same, but the content was differed. Specifically, the SSG13 had stronger intensity peaks than SH18, indicating more new minerals generated than SH18. The reason is that the SS13 activator contains Na_2_SiO_3_ and desulfurized gypsum, and the silicate ions in Na_2_SiO_3_ can polymerize with dissolved Si and Al monomers, which can quickly generate three-dimensional network aluminosilicate geopolymers [23]. Desulfurization gypsum, as a salt activator, can release Ca^2+^ and SO_4_^2−^ ions, which can be adsorbed on the surface of the particles, thus increasing the number of crystal nuclei in the slurry, which is conducive to the volcanic ash effect and improves the strength of the geopolymer [24]. There is unreacted desulfurized gypsum in the XRD spectrum of SSG13, which corresponds to the visible unreacted desulfurized gypsum in the cross section (Figure 2). Since the raw materials of the two geopolymers were coal gangue and GGBFS. Coal gangue is a low-calcium raw material, the primary hydration product is N–A–S–H (Natrium aluminate silicate hydrate) gel [25]. The GGBFS was used as a supplement as the calcium source, mainly in C–S–H (calcium silicate hydrate) and C–A–S–H (calcium aluminate silicate hydrate) crystalline phases [26,27]. It can be seen from the XRD spectrum that the mixture of UCCG and GGBFS formed three kinds of gels after alkali activation, namely N–A–S–H, C–(A)–S–H, and C–N–A–S–H (calcium natrium aluminate silicate hydrate). The N-A-S-H gel includes zeolite minerals such as nepheline, sillimanite, and analcime. The C–A–S–H crystalline phase products were gehlenite, zoisite, and anorthite and the mixture of the two C–N–A–S–H gels produced gonnardite, thomsonite, and mesolite. The color of nepheline, sillimanite, and analcime minerals is green, so the color of the specimen is green (Figure 1 and Figure 2). 

### 3.2. FTIR Tests

**Figure 13:**
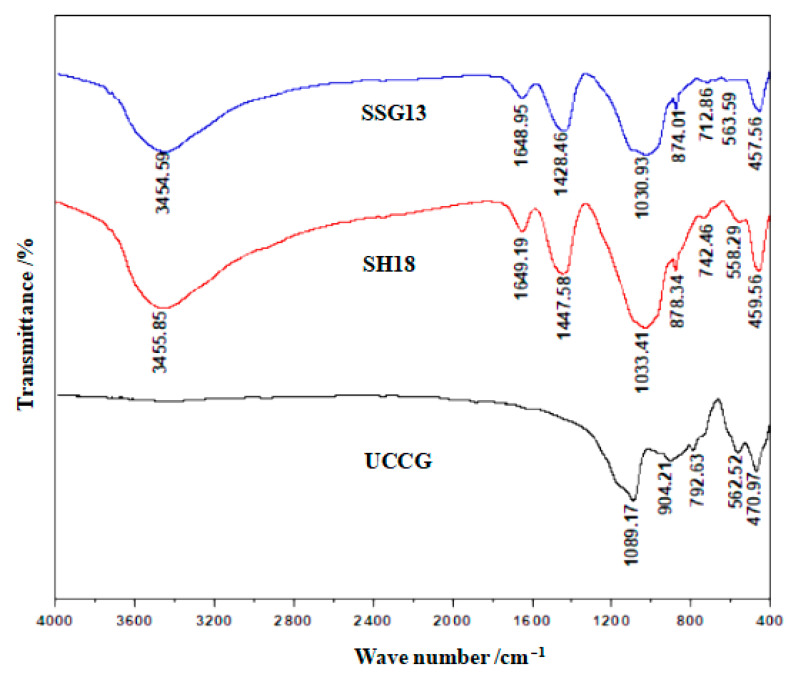
FTIR spectra of geopolymers with different activators.

Figure 13 shows the FTIR tests of the geopolymer. The stretching vibration of the hydroxyl group at 3440–3455 cm^−1^ and the bending vibration of the hydroxyl group at 1644–1649 cm^−1^ of SH18 and SSG13 indicated that there was bound water produced during the polymerization process [28]. The presence of 1428–1447 cm^−1^ was due to the asymmetric stretching pattern of O–C–O bounds in CO_3_^2−^, indicating that the gels were carbonated [29,30]. The fact that SH18 and SSG13 geopolymers had almost identical characteristic lines demonstrates that both geopolymers had similar main structures. In contrast, the distinct spectrum of UCCG powder was confined to a low wave number range. It was observed that the distinct-spectrum wave numbers in UCCG (i.e., 904 and 1089 cm^−1^) were shifted to lower wave numbers of 870 and 1030 cm^−1^ in the two geopolymers. A greater shift in the distinct-spectrum wave numbers was related to a higher degree of the polymerization reaction [31,32]. In comparison, the shift was larger in SSG13 than in SH18, particularly at a lower frequency. This indicates that SSG13 reacted more significantly than SH18, and therefore, generated more geopolymers, which explains why the mixed activator samples developed higher strengths than single activator samples. The characteristic peaks at 457–459 cm^−1^, 558–563 cm^−1^, 712–742 cm^−1^, and 1030–1033 cm^−1^ are the asymmetric stretching vibrations of the Si–O–T bond (T is Si or Al) [33].

### 3.3. SEM and EDS Test

**Figure 14:**
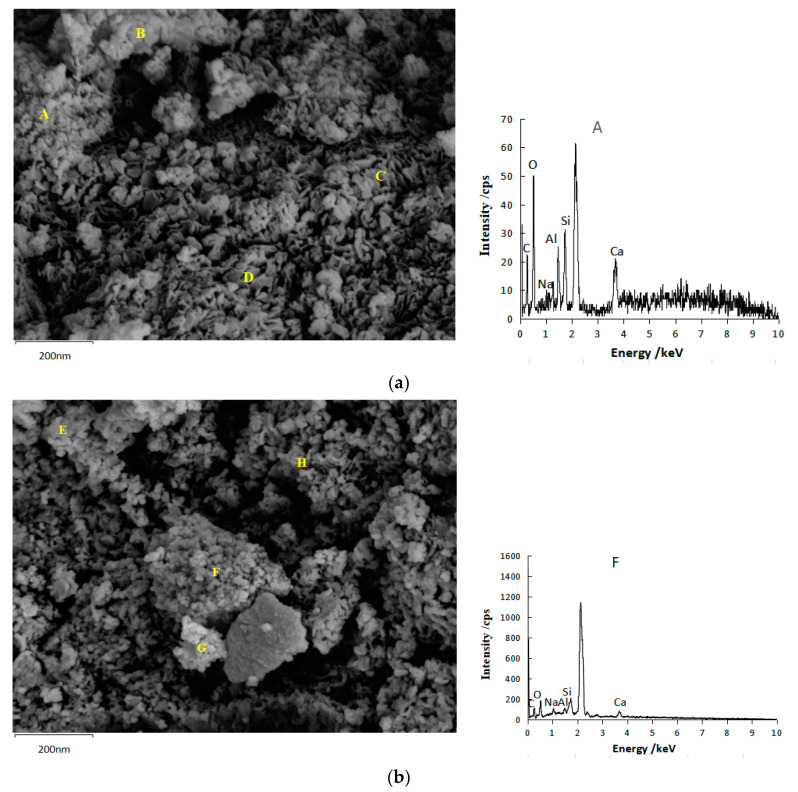
SEM and EDS of geopolymer. (**a**) SH18 geopolymer and A point EDS (**b**) SSG13 geopolymer and F point EDS.

Figure 14 and Table 1 show the SEM and EDS test results of geopolymers. In Figure 14, many cotton flocs, flakes, and flocculent gel substances were produced. Table 1 shows the atomic ratio of each element at the point detected by EDS. Ca, Na, Al, and Si elements were detected at each point, proving that both SH18 and SS13 geopolymers contained C–A–S–H, N–A–S–H, and C–N–A–S–H gels. The Ca/Si ratio of SH18 is above 1, which is much larger than Na/Si and Al/Si, indicating that C–A–S–H gel was the dominant gel in the product [34], accompanied by N–A–S–H and C–N–A–S–H gel. For SSG13, the Ca/Si ratio was lower than 1, and the Na/Si ratio at E, F, and G was 0.3–0.5, which proved that the gels were mainly N–A–S–H or C–N–A–S–H.

## 4. Conclusions

This study proposed a new geopolymer by using ultrafine calcined coal gangue (UCCG) and ground granulated blast furnace slag (GGBFS) as raw materials and varying activators (single or mixed). The UCCG and GGBFS were activated by different activators to form geopolymer gels, the setting time and strength were tested, and the changes of mineral composition, functional group, microscopic morphology, and elements were analyzed. This study provides a theoretical basis for predicting the setting time of coal gangue based geopolymer and rapid configuration of activators in engineering. The following conclusions are drawn from this study.

(1) The setting time of a single activator can be predicted by the molarity of NaOH (y = ax^−b^), and the setting time of a mixed activator can be predicted by the Na_2_SiO_3_ modulus (y = ax^−b^) and the dosage of SS activator (y = ax + b).
(2) The strength value of NaOH and the molar concentration have a quadratic parabola relationship (y = ax^2^ + bx + c), and the NaOH molarity of the corresponding activator at a certain intensity can be calculated by substituting the relational equation. Similar trends are observed for Na_2_SO_3_ modulus and strength in mix activator. Meanwhile, the dosage of SS (NaOH and Na_2_SO_3_) and strength followed a linear relationship (y = ax + b). Therefore, in the mixed activator, the corresponding modulus and the amount of alkali doping at a pre-reaching strength can be calculated through these equations.
(3) To achieve the highest strengths, the optimum mixing ratio in the mixed activator samples was 0.6 modulus, 16% SS, and 6% desulfurization gypsum, the initial and final setting times were 100 min and 325 min, and the 28-day compressive and flexural strengths were 40.1 MPa and 7.8 MPa, respectively. For the single activator samples, the optimum molarity of NaOH was 2 mol/L, the initial and final setting times were 37 min and 47 min, and the 28-day compressive and flexural strength were 23.2 MPa and 7.5 MPa, respectively.
(4) The gangue-based geopolymers are mainly N–A–S–H, C–A–S–H, and C–N–A–S–H gels. The single activator produced more C–A–S–H gels, while the mixed activator generated more N–A–S–H and C–N–A–S–H gels. The mixed activator is recommended in the development of the proposed geopolymers, over that of the single activator, as it yielded higher compressive and flexural strengths. The main reason behind this is that the mixed activator engaged a more intense hydration reaction, therefore producing more cementing gels.

## 5. Materials and Method

### 5.1. Materials

**Figure 15:**
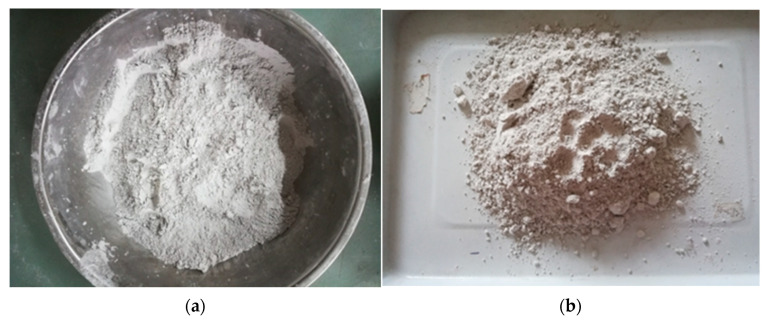
Raw materials. (**a**) UCCG (**b**) GGBFS powder.

**Figure 16:**
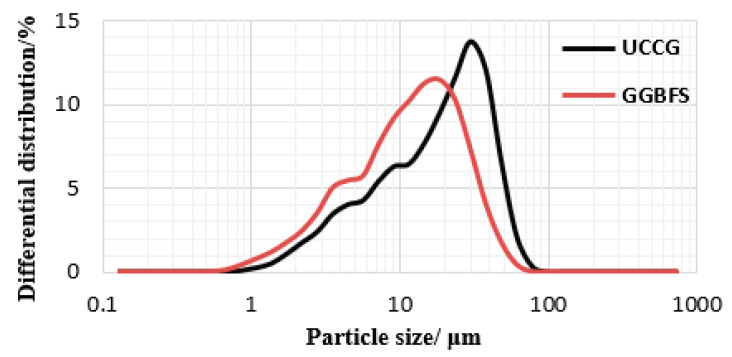
Particle size distribution of UCCG and GGBFS powder.

The main materials (coal gangue and GGBFS powders) and desulfurized gypsum (used to make alkaline solution) were all taken from Hejin, Shanxi Province, China. Prior to geopolymerization, the untreated coal gangue required excitation to enhance its reactivity. Firstly, the coal gangue was mechanically crushed, and then calcined at 700 °C for 2 h in a muffle furnace. The calcined coal gangue was further crushed with an ore crusher and ground with a grinding machine to make ultrafine calcined coal gangue powder (UCCG). The GGBFS powder used was grade S95. Figure 15 shows the photographs of UCCG and GGBFS powders. The particle sizes of UCCG and GGBFS were measured by laser particle size analyzer (LS-POP (9), OMEC, Zhuhai, China). The particle size distribution and results are shown in Figure 16 and Table 2. D50, D (4, 3), and D (3, 2) are the average particle size, volume average particle size, and surface area average particle size, respectively. Therefore, UCCG and GGBFS powder belong to ultrafine powders. Table 3 shows the chemical composition of UCCG and GGBFS. One activator consisted of only NaOH, which was analytically pure with ≥96% concentration while the other activator consisted of NaOH, liquid Na_2_SiO_3_ (SiO_2_ content is 29.99%, Na_2_O content 13.75%, the Baume 50Be.), and desulfurized gypsum (CaSO_4_·2H_2_O ≥ 93%). 

### 5.2. Methods

#### 5.2.1. Mix Design Considering Effects of Activators

##### Mixing with a Single Activator

The percentages of GGBFS powder relative to the dry weight of the samples were set to 10%, 20%, 30%, and 40%. In each GGBFS dosage, the single activator (i.e., NaOH) with molar concentrations of 1, 1.5, 2, 2.5, and 3 mol/L was added to make five test samples as shown in Table 4 where SH denotes a single activator. As such, a total of four groups and 20 test samples were prepared to evaluate the effect of a single activator on the sample strength development. 

##### Mixing with Mixed Activator

The mixed activator was composed of NaOH, Na_2_SiO_3_, and desulfurization gypsum, denoted with SSG. Sodium hydroxide and sodium silicate are denoted with SS. In this part of the experiment, the GGBFS powder dosage was kept at 40%, UCCG dosage was 54%, the dosage of desulfurized gypsum was 6%, and the SS alkali content was 10%, 12%, 14%, and 16% of the total amount of UCCG and GGBFS. Under each SS dosage, four numbers of Na_2_SiO_3_ modulus were considered as tabulated in Table 5.

#### 5.2.2. Geopolymer Gels Preparation and Tests Method

**Figure 17:**
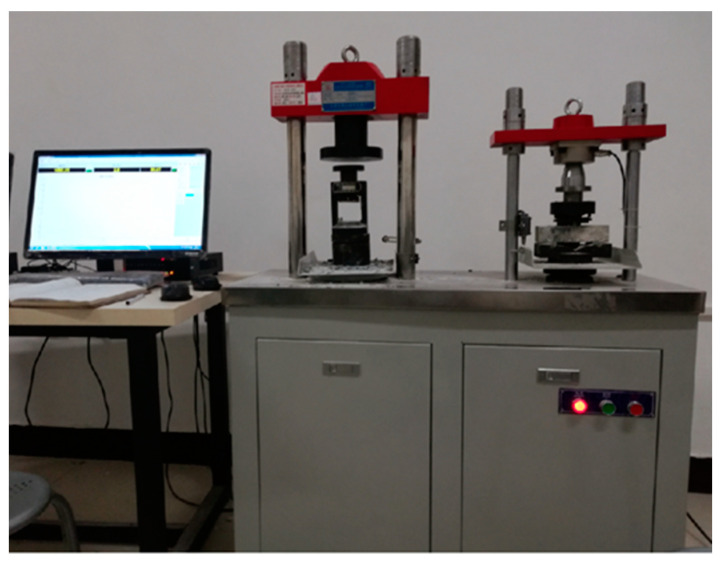
Automatic compressive and flexural testing machine.

The activator (i.e., single or mixed) was added with deionized water in the proportions specified in Section 2.2.1 and stirred with a magnetic mixer for 30 mins. The ratio of liquid to binder was 0.45 (m_Liquid_ = m_water_ + m_water in N__a_2_SiO_3__. m_binder_ = m_UCCG_ + m_GGBFS_). The alkaline solution was then added simultaneously with the UCCG and GGBFS powders and mixed in a cement mixer according to the standard mixing method: i.e., mixing for 120 s at a low speed (The revolution of the stirring blade is 62 r/min, and the self-conversion is 140 r/min), holding for 15 s, and the mixing again 120 s at a high speed (The revolution of the stirring blade is 125 r/min, and the self-conversion is 285 r/min). After that, a portion of the geopolymer slurry was used to determine the setting time, another part of the slurry was poured into a 40 × 40 × 160 mm mold, which was then subjected to vibration 60 times on the vibration platform. After vibration, the slurry was levelled and covered with plastic, and then transported to a standard curing box with a temperature of (20 ± 2) °C and relative humidity of ≥95% [35]. After 24 h of curing, the mold was dissembled while the sample continued to be cured to the prescribed times of 3, 7, and 28 days. The test instrument is an automatic compressive and flexural testing machine (WAY-300B.Wuxi Xiyi Building Material Instrument Factory, China, Figure 17). The size of the flexural specimen was 40 × 40 × 160 mm, the flexural strength was the average of three specimens, the side length of the flexural fixture was 40 mm, the span is 100 mm, and the loading speed was 50 N/s. The compressive specimens were the specimens broken after the completion of the flexural test, and the final strength of the specimen was the average of the compressive strength of the six test blocks. The compression clamp was 40 × 40 mm, and the loading speed was 2.4 kN/s. Following the strength tests, 28-day samples were further used for mineral and microstructure analyses. Specifically, the samples were ground and soaked in anhydrous ethanol for 24 h to terminate hydration. After that, the samples were then placed in an oven to dry and stored in an airtight bag at room temperature (25–28 °C). XRD test was performed with the scanning angle of 5°, −90°, and 8° per minute using Model D8 Advance, Bruker AXS, Germany. FITR (Thermo Scientific Nicolet iS5, The United States) was utilized to perform FITR analyses to determine changes in functional groups and chemical bonds of the geopolymer samples. The microscopic morphology and elements were analyzed by SEM and EDS (SEM is Zeiss Sigma 300, Germany. EDS is Oxford X-Max, UK).

## Figures and Tables

**Table 1:** Atomic percentage at each point in the EDS test/%.

Point	Ca	O	C	Na	Al	Si	Ca/Si	Al/Si	Na/Si
A	9.78	47.14	30.05	0.93	4.86	7.24	1.35	0.67	0.13
B	13.79	44.94	28.32	3.15	3.51	6.29	2.19	0.56	0.50
C	10.77	48.52	23.65	3.24	5.38	8.44	1.28	0.64	0.38
D	14.61	43.67	14.61	1.97	4.82	7.79	1.88	0.62	0.25
E	5.45	28.13	55.11	2.54	1.86	6.57	0.83	0.28	0.39
F	8.10	36.55	41.11	3.15	2.62	8.45	0.96	0.31	0.37
G	3.50	24.38	64.66	1.96	2.00	3.50	1.0	0.57	0.56
H	7.00	29.13	49.31	1.17	3.58	9.81	0.71	0.36	0.12

**Table 2:** Particle size analysis results/µm.

Materials	D10	D50	D90	D97	D100	D(3,2)	D(4,3)	Span
UCCG	3.647	17.157	37.319	47.252	76.767	8.838	19.154	1.963
GGBFS	2.755	10.687	26.483	36.201	60.654	6.167	12.945	2.220

**Table 3:** Chemical composition of UCCG and GGBFS/%.

Materials	SiO_2_	Al_2_O_3_	Fe_2_O_3_	CaO	MgO	SO_3_	TiO_2_	K_2_O	Na_2_O	Others
UCCG	54.2	41.6	0.98	0.55	0.10	0.02	0.96	0.91	0.46	0.22
GGBFS	34.9	16.7	1.05	33.5	6.0	1.7	-	-	-	6.15

**Table 4:** Single activator mix ratio design table.

**Sample No.**	**SH1**	**SH2**	**SH3**	**SH4**	**SH5**	**SH6**	**SH7**	**SH8**	**SH9**	**SH10**
GGBFS/%	10	10	10	10	10	20	20	20	20	20
C_NaOH_/mol·L^−1^	1.0	1.5	2.0	2.5	3.0	1.0	1.5	2.0	2.5	3.0
**Sample No.**	**SH11**	**SH12**	**SH13**	**SH14**	**SH15**	**SH16**	**SH17**	**SH18**	**SH19**	**SH20**
GGBFS/%	30	30	30	30	30	40	40	40	40	40
C_NaOH_/mol·L^−1^	1.0	1.5	2.0	2.5	3.0	1.0	1.5	2.0	2.5	3.0

**Table 5:** Mixed activator ratio test table.

**Sample No.**	**SSG1**	**SSG2**	**SSG3**	**SSG4**	**SSG5**	**SSG6**	**SSG7**	**SSG8**
SS alkali/%	10	10	10	10	12	12	12	12
Na_2_SiO_3_ modulus	0.6	0.8	1.0	1.2	0.6	0.8	1.0	1.2
**Sample No.**	**SSG9**	**SSG10**	**SSG11**	**SSG12**	**SSG13**	**SSG14**	**SSG15**	**SSG16**
SS alkali/%	14	14	14	14	16	16	16	16
Na_2_SiO_3_ modulus	0.6	0.8	1.0	1.2	0.6	0.8	1.0	1.2

## Data Availability

Data obtained as described.
